# A qualitative examination of primary care team’s participation in the distribution of the COVID-19 vaccination

**DOI:** 10.1186/s12875-024-02327-2

**Published:** 2024-03-14

**Authors:** Rachelle Ashcroft, Catherine Donnelly, Simon Lam, Peter Sheffield, Bryn Hamilton, Connor Kemp, Keith Adamson, Judith Belle Brown

**Affiliations:** 1 Factor-Inwentash Faculty of Social Work, University of Toronto, 246 Bloor Street West, Toronto, ON M5S 1V4 Canada; 2 Faculty of Health Sciences, Queen’s University, Kingston, ON Canada; 3 Association of Family Health Teams of Ontario, Toronto, ON Canada; 4 Department of Family Medicine, Schulich School of Medicine and Dentistry, Western University, London, ON Canada; 5 Frontenac, Lennox, and Addington Ontario Health Team, Kingston, Canada

**Keywords:** Primary health care, Interprofessional, Vaccinations, COVID-19 vaccination distribution

## Abstract

**Background:**

Primary health care (PHC) has historically led and implemented successful immunization programs, driven by strong relationships with patients and communities. During the COVID-19 pandemic, Canada began its vaccination strategy with mass immunizations that later included local efforts with PHC providers. This study seeks to understand how PHC contributed to the different phases of the COVID-19 vaccination rollouts in Ontario, Canada’s most populous province.

**Methods:**

We conducted a descriptive qualitative study with focus groups consisting of PHC providers, administrators, and staff in Ontario. Eight focus groups were held with 39 participants representing geographic diversity across the six Ontario Health regions. Participants reflected a diverse range of clinical, administrative, and leadership roles. Each focus group was audio-recorded and transcribed with transcriptions analyzed using thematic analysis.

**Results:**

With respect to understanding PHC teams’ participation in the different phases of the COVID-19 vaccination rollouts, we identified five themes: (i) supporting long-term care, (ii) providing leadership in mass vaccinations, (iii) integrating vaccinations in PHC practice sites, (iv) reaching those in need through outreach activities; and (v) PHC’s contributions being under-recognized.

**Conclusions:**

PHC was instrumental in supporting COVID-19 vaccinations in Ontario, Canada across all phases of the rollout. The flexibility and adaptability of PHC allowed teams to participate in both large-scale and small-scale vaccination efforts.

**Supplementary Information:**

The online version contains supplementary material available at 10.1186/s12875-024-02327-2.

## Background

An important step towards recovery from the COVID-19 pandemic is mass distribution of a vaccine [1–4]. Primary health care (PHC) teams - comprised of family physicians and/or nurse practitioners working in partnership with other healthcare providers - are well positioned to provide leadership in the dissemination of the COVID-19 vaccine due to a wide range of expertise and experience with vaccinations [4, 5].

### Canada’s COVID-19 vaccination roll-out

The WHO declared a Public Health Emergency of International Concern due to the spread of the novel coronavirus – COVID-19 – on January 30, 2020 [6]. By the end of December 2020, Pfizer/BioNTech, Oxford and AstraZeneca had reported the development of effective COVID-19 vaccinations [7–9]. While the federal government of Canada was responsible for procuring COVID-19 vaccines, the provincial and territorial governments distributed and administered vaccinations to residents within their jurisdictions [10] through a phased rollout [11–13]. A complete detailed breakdown of the vaccination rollout across Canada is available from CIHI [14].

### Role of PHC in the distribution of the COVID-19 vaccination

PHC is the first point of access in the healthcare system and has a long history of successfully delivering immunization programmes [15–20]. According to Hogg & Johnston [5], the vaccination system is built on counselling and vaccine infrastructure provided by PHC. The successful delivery of millions of influenza immunizations every year, and longstanding success in operationalizing the childhood vaccination programme, make PHC optimally positioned to deliver COVID-19 vaccines [4]. PHC has well-established and trusting relationships with patients and communities, which provides knowledge of local population and an avenue for timely vaccinations [4, 5]. Further, PHC is well-placed to enhance equitable access and vaccine uptake [4]. With expertise in education and health promotion related to vaccinations [4], PHC may be in a unique position to address COVID-19 vaccine hesitancy.

### Study rationale

Although there are some emerging reports on the experiences of family physicians [5], the role that interprofessional PHC teams have played in the distribution of the COVID-19 vaccination has not been studied to our knowledge, and there is a paucity of empirical evidence detailing PHC’s involvement in the distribution of the COVID-19 vaccination [7]. Team-based PHC can play a unique role in vaccination programs, including COVID-19 vaccination. Our study sought to understand the phases of the COVID-19 vaccination strategy that interprofessional PHC teams in Ontario participated in. Understanding how PHC teams have contributed to the distribution of the COVID-19 vaccination will be critical to informing policy and practice decisions about future COVID-19 boosters and other vaccinations.

## Methods

### Design

The study used a qualitative descriptive research design which guided sampling, data collection using focus groups, and data analysis [21]. Research team members had a range of clinical, leadership, and research expertise with disciplinary backgrounds representing social work, occupational therapy, family medicine, and health services research. Research was conducted in partnership with the Association of Family Health Teams of Ontario (AFHTO), a provincial organization that provides leadership and advocates on behalf of PHC teams in Ontario.

### Setting

In Ontario - Canada’s most populous province with 14.7 million residents [12] - the COVID-19 vaccination program began on December 14, 2020 [13]. Family Health Teams are one model of team-based PHC in Ontario [22] and are recognized as a patient medical home [23]. At the time of our study, there were 184 Family Health Teams in Ontario [24] which is the largest team-based PHC model in Canada. Family Health Teams may be comprised of family physicians, nurse practitioners, nurses, social workers, pharmacists, dietitians, as well as other types of healthcare providers [22]. The size of each Family Health Team organization, as well as the types and number of services provided by the family physicians and other interprofessional providers vary depending on population size and composition [25].

### Sample and recruitment

We used a purposive sampling technique to engage a diversity of perspectives from various healthcare providers that work in Family Health Teams and played a role in the distribution of the COVID-19 vaccination. Potential participants were those working in Family Health Teams who had played a role in the support and distribution of the COVID-19 vaccination and were able to participate in an online focus group. Our aim was to recruit healthcare providers representing Family Health Teams from each of the six Ontario Health regions: West, Central, Toronto, East, North West, and North East [26]. We strived for representation from each of these six regions to (i) include regional variation in terms of rural and urban; (ii) reflect the varying diversity of populations in these regions; and (iii) gain a provincial-wide understanding. We recruited healthcare providers working in Family Health Teams by emailing Executive Directors and inviting them to share our recruitment materials with members of the team. Additionally, AFHTO distributed the recruitment information widely by sending emails to the provider community of practice listservs. Recruitment was also done using social media (i.e., Twitter), and was advertised in the AFHTO newsletter. This broad recruitment strategy meant that healthcare providers from across all 184 Family Health Teams were invited to participate in the study. Recruitment materials provided an overview of the study goals, information about the principal investigator including contact information, and confirmation of research ethics approval. Potential participants contacted the study coordinator (SL) by email to express interest in participating in a focus group.

### Data collection

As a research team, we collaboratively developed a semi-structured interview guide (see supplementary file) informed by emerging literature on PHC and the COVID-19 pandemic, and our teams’ previous research with team-based PHC. Data was collected using focus groups conducted via an online virtual video platform, with an aim of four to six participants per focus group as is recommended for online focus groups [27]. We used focus groups for data collection because this dynamic method enables a process to understand providers’ perspectives that arise during live discussions [28, 29]. Focus groups were conducted with providers who had similar disciplinary backgrounds and lasted 60–90 min in duration. The rationale for conducting focus groups organized around provider groups was to minimize power differentiation in order to promote maximum engagement in the focus group discussion. Two research team members co-facilitated each of the focus groups (RA/CD and/or SL). Focus groups were audio-recorded and transcribed verbatim immediately following the interview. Participants were randomly assigned a code for confidentiality. Interviewers made field notes immediately following each focus group.

### Data analysis

Data collection and data analysis occurred concurrently, and data were analyzed using thematic analysis [30, 31]. One researcher was assigned the primary data analyst (PS) while two other researchers (RA/SL) acted as secondary analysts. Primary and secondary data analysts first became familiar with the data by reviewing the transcripts. Once familiar with the data, the primary data analyst generated initial codes in the data that were reviewed by the secondary coders. During the process, the primary and secondary coders met weekly. In qualitative research, the quality of the data is determined by various strategies including conducting individual and team analysis. During team analysis, the team members shared and discussed their coding of the data to ensure that their interpretation of the data aligned. When the initial coding was complete, the primary and secondary data analysts then met to confirm coding structure with the data analysis sub-committee comprised of the primary and secondary data analysts plus two other research team members (CD/JBB). The data analysis sub-committee reviewed the codes for potential themes. Subsequently, the data analysis sub-committee named and defined the themes derived from the data. During this process, we identified exemplar quotes to illustrate key themes. Then, all members of the research team had an opportunity to review themes and provide input on the interpretation of the data. Finally, we related themes and codes back to the study aims through process of manuscript preparation. Data saturation was reached [30]. Rigour and trustworthiness was achieved through reflexivity, prolonged engagement, and peer debriefing [31]. We used NVivo12 to help organize the data and facilitate the analysis process.

## Results

**Fig. 1:**
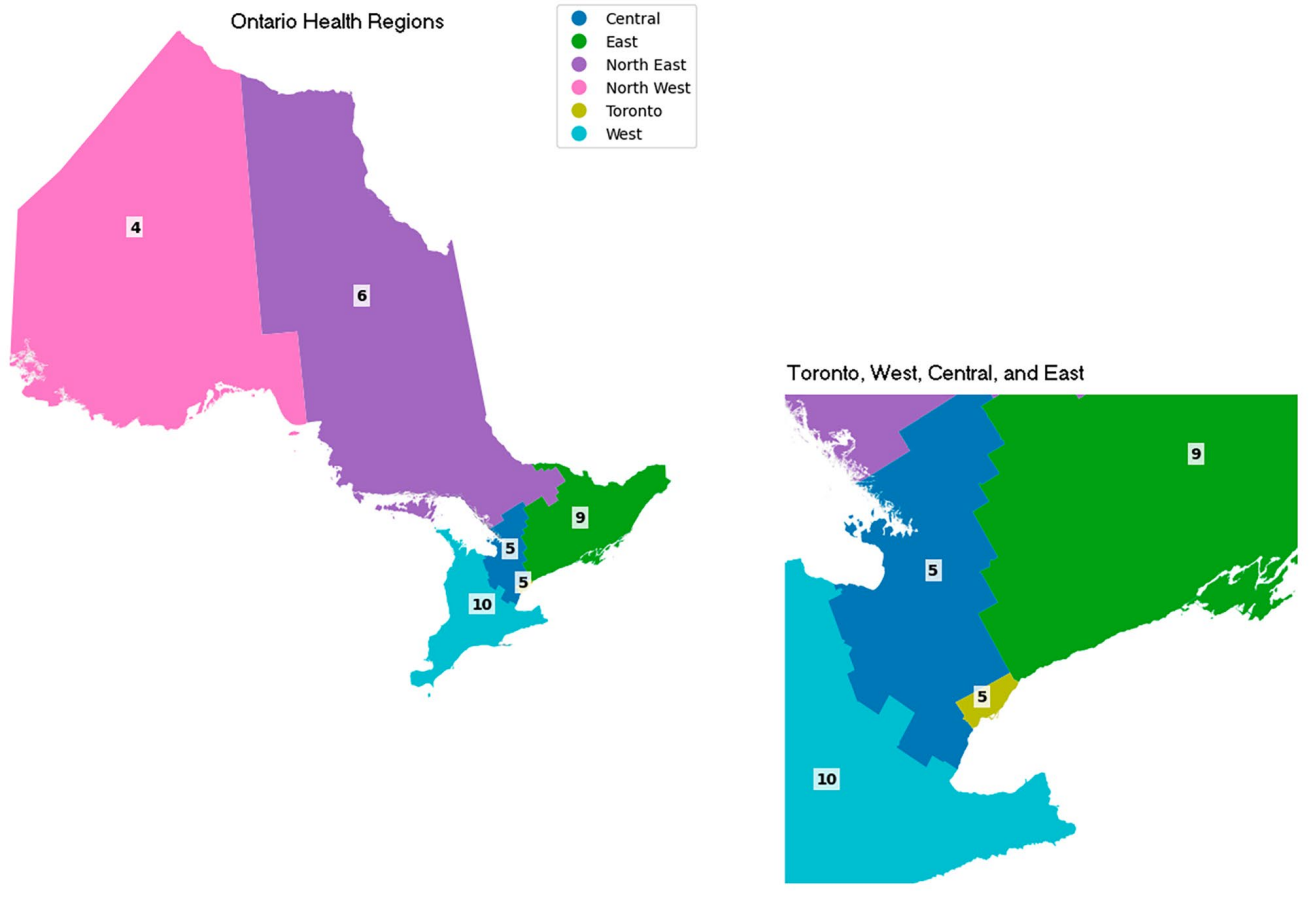
Number of participants in each of the six health regions of Ontario, Canada (N = 39)

A total of 39 participants were included in eight focus groups. Focus groups were organized based on provider groups (i.e., physicians, nurses, executive directors, quality improvement decision support specialists) when possible; however, we conducted 3 focus groups comprised of interprofessional healthcare providers (IHPs) when we did not have enough participants to run provider-specific focus groups. The largest focus group included six participants, although there was an overall average of five participants per focus group. The participants (*N* = 39) represented geographic diversity across each of the six Ontario Health regions West (*n* = 10), East (*n* = 9), North East (*n* = 6), Central (*n* = 5), Toronto (*n* = 5), and North West (*n* = 4). Figure 1 illustrates a map indicated the number of participants in each of the six health regions of Ontario Canada.

In terms of geographical representation, there was a similar representation of participants from urban (*n* = 21), rural (*n* = 16), and mixed (*n* = 1) settings, with *n* = 1 unknown. Table 1 provides an overview of each of the 8 focus groups including types of providers involved in each focus group, participant composition, and numbers of participants.

**Table 1:** Overview of eight focus groups including type of provider group, participant numbers, and participant composition (*N* = 39)

Focus Group #	Types of Providers	Participant Composition	# of Participants
**1**	Executive Directors	6 Executive Directors	6
**2**	Quality Improvement Decision Support Specialists	5 Quality Improvement Decision Support Specialists	5
**3**	Nurses	3 Nurses 2 Nurse Practitioners	5
**4**	Nurses	3 Nurses 1 Nurse Practitioner	4
**5**	IHPs	2 Health Promoters 1 Clerical Staff 1 Pharmacist 1 Respiratory Therapist	5
**6**	IHPs	2 Clerical Staff 2 Nurses	4
**7**	IHPs	3 Pharmacists 1 Dietitian 1 Nurse Practitioner	5
**8**	Family Physicians	5 Family Physicians	5
**TOTAL**			**39**

With respect to understanding PHC teams’ participation in the different phases of the COVID-19 vaccination rollouts, we identified five themes: (i) supporting long-term care, (ii) providing leadership in mass vaccinations, (iii) integrating vaccinations in PHC practice sites, (iv) reaching those in need through outreach activities; and (v) PHC’s contributions being under-recognized.

### Supporting long-term care

Family physicians, holding multiple roles in their communities, explained that they were instrumental in facilitating the vaccination roll-out in long-term care facilities: “*A few of us in [name of city] approached Public Health, to find out if they needed help getting into long-term care homes*” (FG8, P5, Physician). Similarly, “*With regards to long term care, we facilitated getting doses over to long-term care homes, all of us who work on the Family Health Team are the medical directors of long-term care home*” (FG8, P3, Physician). The focus group of Quality Improvement Decision Support Specialists (QIDSS) shared, “*We started with long-term care, so we had clinics set-up for long-term care residents and…the people working there first*” (FG2, P4, QIDSS).

PHC provided a range of human resources to assist with vaccinations in long-term care. As explained by a respiratory therapist, “*You just basically tell the ED and supervisor what day you prefer…there’s only few went to the long-term care, mostly…it’s the nurses, nurse practitioners and the doctor going*” (FG5, P1, Respiratory Therapist). Participants representing rural communities highlighted the essential role that PHC teams played in all phases of the vaccination rollout, including in long-term care. According to the focus group of QIDSS: “*I’d say for some of the more rural [Family Health Teams] where they are the only shop in town, I mean, they were definitely more involved in all aspects of the rollout. So in long-term care, they helped with the rollout there*” (FG2, P2, QIDSS).

Not all PHC teams participated in long-term care vaccinations. Some participants indicated that members of their PHC team were not involved with the vaccination distributions in long-term care: “*We didn’t do much in long-term care. That was mostly driven by Public Health*” (FG8, P4, Physician). A nurse practitioner participant noted: “*with our team specifically I don’t believe we helped out at long-term care*” (FG3, P4, Nurse Practitioner).

### Providing leadership at Mass Vaccination

In all the focus groups, PHC leadership pertaining to the organizing and operationalizing of mass vaccination efforts was a prominent theme. Focus groups described at length the engagement PHC providers and teams had in mass vaccination clinics: “*The Family Health Team, we’ve done all of the mass immunization clinics, the entire time*” (FG5, P1, respiratory therapist). Similarly, a family physician noted:

*Mainly driven by the county and the Family Health Team, we set up a mass vaccination clinic in our largest town center….we organized and ran mass clinics there from April 2021 onward… with the new wave…it and now it’s back up and running, delivering over 26,000 doses in our rural community* (FG8, P3, Physician).

Similarly, the focus group comprised of nurses and nurse practitioners highlighted the leadership role of PHC in organizing and running mass vaccination clinics: “*In January…we actually set up and ran our own mass clinics at one of the local sports centers and we did that, over the course of a two and a half week period, doing it every day except for Sundays*” (FG3, P1, Nurse Practitioner). Some mass vaccination clinics were run at the PHC site itself, yet extended to all members of the community: “*We’re rural, and so what we brought to the table was, we did mass clinics out of our site, and so we expanded the access to the rural communities out here, and not all people were patients of ours, they were just across the community*” (FG1, P1, Executive Director). Participants in thehe IHP focus group noted, “*We did large scale drive through clinics, we kind of repurposed our vaccine committee to replan how a drive through clinic would work*” (FG5, P4, Health Promoter).

In all the focus groups, there were participants who talked about how their PHC team contributed to mass vaccination clinics run in partnership with public health and other local organizations:

*We had 12 immunizers, 1 physician for monitoring of patients with AEs [Adverse Events], approximately 4–6 pharmacy students, pharmacists, technicians for vaccine preparation, 5–10 staff members for check-in/out, and multiple volunteers to guide patients through the clinic and monitor after receiving the vaccine. Staffing was a strength of our clinic given the relationship with the Family Health Team*. (FG5, P3, Pharmacist)

Running the mass vaccination clinics required significant human resources, with some of the human resources provided by PHC as explained in the QIDSS focus group: “*In terms of mass vaccination…Public Health asked the Family Health Team to join in the effort, to…participate in mass vaccination…some of the staff was seconded to do that*” (FG2, P1, QIDSS). Across all focus groups, there were participants who spoke about the contributions that PHC made by staffing the mass vaccination clinics: “*Within the Family Health Team we have nurse practitioners, we have pharmacists and registered nurses, and RPNs…and so a lot of us did…get called upon to help out at the mass vaccination clinics in [city name]*” (FG3, P4, Nurse Practitioner). The nurse focus group also noted the role that PHC played in staffing the mass vaccination clinics: “*We have 15 physicians on staff, so our physicians went out to the arena, and vaccinated there, from probably April to…November 2021. We had some staff go vaccinate as well, some nurses who are seconded from the [Family Health Team]and went over to vaccinate a couple days here and there*” (FG4, P3, Nurse). Several focus groups highlighted that the PHC nurses played a particularly central role in administering the vaccinations: “*The mass clinics…we had a pharmacist helping draw up, but basically it was always the nurses and the NPs…primarily providing the shots”* (FG6, P2, clerical staff). PHC contributed a range of staff to support the operations of mass vaccination clinics in their communities, including clerical staff as explained by in the focus group of executive directors: “*Public health was actually doing the process, doing the ordering, online booking…was a nightmare, so we asked if we could help by…giving some of my reception staff who are working evenings, they actually got the rights to actually do booking into the booking clinics of public health because they were overwhelmed*” (FG1, P5, Executive Director). The family physician focus group also highlighted the staffing contributions to the mass vaccination clinics:

*We decided to…donate time to the mass clinic…at the…community center…We spoke to the physician that…was running the clinic and we basically said, ‘How many tables are you going to need at each time?’ Turned out it was 14, each table having one vaccinator. We would give two tables consistently throughout the rollout. So if we had 14 tables required, two of them would come from our Family Health Team*” (FG8, P5, Physician).

Some PHC teams opted to participate in mass vaccination clinics instead of hosting vaccinations on-site due to their own space limitations: “*Starting in March of 2021, we started with the mass clinics because we just didn’t have space here to do the numbers*” (FG1, P3, Executive Director). It is important to note that not all PHC teams participated in the mass vaccination clinics. One challenge that prevented some teams from participating had to do with a lack of PHC providers: “*I’m just very impressed that some of the teams were able to supply staff seconded to mass vaccination clinics…I have more rural teams and there were just no staff to send out, like it was just impossible*” (FG2, P3, QIDSS).

### Integrating vaccinations in PHC practice sites

In all focus groups, integrating vaccinations within PHC practice sites was a prominent theme:

*In November of 2021 we were asked to start vaccinating in-clinic, when the big push for getting people vaccinated came along, so we started doing vaccination clinics here. We vaccinated…close to 3000 people so far in our clinic, both patients and non-patients…we vaccinate all populations, we can do boosters. We do it all* (FG4, P3, Nurse).

Some vaccination clinics made the COVID-19 vaccination available to their patients as well as members of the public: “*What the Family Health Teams did were…scheduled clinics. As the mass vaccination clinics were winding down the Family Health Teams were asked to take up the slack, and they actually scheduled clinics, not just for their patients but also for the general public.*” (FG2, P3, QIDSS). The family physician focus group discussed implementing vaccination endeavours at their practice sites: “*For our Family Health Team, we have a clinic of four docs…so we did run full pop-up clinics and did most of our population here*” (FG8, P1, Physician). One of the Family Health Teams offered drive-through vaccinations to patients to enhance patient safety: “*Our Family Health Team ran some drive-through vaccination clinics, which were very successful, and patients absolutely loved it - especially the older folks… So that was a lot of the focus of our Family Health Team, in terms of getting our own patients done*” (FG8, P4, Physician). A medical receptionist in a different focus group noted, “*We evolved to where we were offering clinics within our clinics…and that worked out well for us because of the patients…it was easier for them to come here*” (FG5, P2, medical receptionist). She further elaborated by saying, “*We’ve done now 45 clinics since June and our nurse practitioner, she ran it…and we didn’t have to go outside of the building for anything…we just did it and away we went and did the best we could*” (FG5, P2, Medical Receptionist). Similarly, the executive director focus group noted:

*[We were] trying to serve those whose needs weren’t…best met in a mass clinic type site…we had private rooms, we had primary care nurses and physicians on-site, we also offered pure counselling appointments, so people that weren’t quite ready for the shot yet they could do either in-person, drop-in, or telephone counselling only to try to address any hesitancy* (FG1, P4, Executive Director).

The nurse focus group indicated that regularly scheduled vaccination clinics occurred at her site: “*for first and second doses…we just concentrated on our patients here at the Family Health Team*…*we ran clinics Monday to Friday alongside the regular clinic that was running* (FG3, P2, Nurse). There were variations in the vaccination initiatives across the various PHC teams because of space restrictions: The…*Family Health Team…had run a drive-through before, so they had a really well-oiled system to do that…but some other teams, some other teams it was maybe less volume…and some teams downtown it was not drive-through because they did not have a parking lot* (FG2, P1, QIDSS). Across focus groups, participants explained that the vaccine initiatives were aimed at their patients as well as the general community:

*Those targeted emails that we send out, we really only administered vaccines to patients of our clinic. We have about 20,000 patients…so we felt kind of accountable for serving our patients needs first. The only time that we really reached out outside of our clinic was when the clinics weren’t filling up, and so there’s an elementary school right across the street, so we went to the administrators, and said if there’s any teachers, if there are staff that want to come to the clinic. And then we also went to the local pharmacy who we knew was maintaining a waitlist, and said, if anyone on your waitlist wants to come to our weekend clinic*. (FG5, P4, Health Promoter)

PHC facilitated opportunistic vaccination, as described by the focus group with QIDSS: “*There were opportunistic doses of vaccines given out - this was when people with COPD or something like that came in for a visit for their chronic condition, and they were just vaccinated if they weren’t vaccinated*” (FG2, P3, QIDSS). The focus group of executive directors showcased the importance of integrating opportunistic vaccinations that facilitated patient-centred access that aligned with the patients’ readiness for a vaccination:

*We were allowed to have supply on hand…when the physicians would have someone in that was anxious about the COVID vaccine, they would chat with them and then all of a sudden they’d say, ‘Well, if you had a dose, I would maybe take it’…and then they’d run down the hallway, and we’d get them vaccinated right while they’re here* (FG1, P1, Executive Director).

Similarly, participants noted that PHC was efficient by integrating the COVID-19 vaccination with the larger vaccination framework. We were encouraged to give other vaccines with the COVID vaccine. So we gave lots of flu shots as well. We would offer flu shots. A (FG4, P4, Nurse Practitioner). For some teams, this approach facilitated the commitment to routine PHC. The family physician focus group noted, “*We felt very strongly that was a good contribution, and that it allowed us to maintain our office practices of seeing patients, and not get bogged down by doing the vaccine. So that’s how our [Family Health Team] did it*” (FG8, P5, Physician). The executive director focus group also highlighted that COVID-19 vaccinations provided an opportunity to do opportunistic vaccinations for the flu: “*Our building is big enough to probably do 80 people on a Wednesday afternoon and we were able to do flu shots at the same time, so we thought this would be an opportunity to do both, we jabbed you on both sides and that worked well*” (FG1, P5, Executive Director). Across focus groups, participants explained the commitment that PHC had to ensure that patients had access to the COVID-19 vaccinations: “*The doctors themselves…they wanted to make sure we had vaccine available for our patients*” (FG6, P2, Clerical Staff).

Some participants noted that not all PHC practices distributed vaccinations but still contributed by supporting patients in booking appointments. In a focus group of nurses, a participant shared that even though vaccinations were not being done at her PHC clinic, the team was instrumental in helping patients obtain access to vaccinations: “*We weren’t doing vaccines clinics at those times. We would spend a lot of time trying to do that for them. So searching for openings…we spent a lot of time doing the searching and booking*” (FG4, P2, Registered Nurse). Not all PHC teams opted to distribute the COVID-19 vaccination, as explained in the family physician focus group:

*There was a lot of noise throughout the pandemic about whether family physicians should be doing this in their offices. There were a lot of people that were very pro, there were a lot of people that were against. Wherever you landed was up to you…Our [Family Health Team] … decided not to do it, simply because we didn’t feel it could be done … because of the…amount of time you had to put people into COVaxx. And also you had to do a lot of people because once you open up the bottle of vaccine, it can only stay for so long *(FG8, P5, Physician).

### Reaching those in need through outreach activities

Outreach activities was a promintent theme. One of the outreach efforts extended to schools: “*We’ve done a few of them in the schools. We did - we have two high schools - so we did some clinics at the high schools, we’ve gone to the elementary schools*” (FG5, P1, Respiratory Therapist). Similarly, “*We ran a special three-day…vaccination event for teachers and daycare workers where we called all the schools and they booked their teachers - schools and daycare workers - and they brought their families*” (FG1, P4, Executive Director).

Within all focus groups, participants explained that members of PHC teams did outreach to ensure vaccinations reached homebound persons: *“[We] also did home visits for the few people that could not get in*” (FG1, P6, Executive Director). The QIDSS focus group also highlighted outreach activities: “*The Family Health Teams also conducted outreach to patients who were at home…who couldn’t get into the clinic, potentially because of COVID issues, but also because they were so seriously ill they were homebound*” (FG2, P3, QIDSS). In an IHP focus group, outreach was mentioned: “*Some of our physicians did home visits to immunize their homebound patients*” (FG5, P5, Health Promoter). The family physician focus group emphasized outreach activities for homebound persons, “*We also created a homebound vaccination program taking the vaccine to homebound patients of all ages*” (FG8, P2, Physician). Although not all teams engaged in outreach to homebound patients, “*We had less access to do home-based vaccinations. It was one of the frustrations that a lot of us had [about] some homebound seniors. We had no idea how we were going to get to them*” (FG8, P5, Physician).

PHC also conducted outreach activities to distribute COVID-19 vaccinations to unhoused persons. As described in the nurse focus group, “*Shelter clients were one of the first populations after kind of frontline healthcare, I think, back in February 2021, started doing shelters. So our team was helping with that*” (FG4, P2, Registered Nurse). While other teams conducted outreach activities to support a range of specialty clinics that required extra support:

*We also did some…cancer clinics specialty clinics, either mobile or on site just on demand, in partnership when public health was just exceeding their capacity…they reached out to us and said, ‘Can your team go out and support some of those other populations?’* (FG1, P4, Executive Director).

Some outreach activities targeted areas with lower vaccination rates, as explained in the family physician focus group: “*We even took a bus out…and we took it along some areas that were lower in vaccination rates, and took our bikes…and just tried to drop in on some establishments, letting them know where and when we would be vaccinating. So really trying to be as patient or person-centered as possible*” (FG8, P2, Physician). Some outreach activities targeted areas most frequented by the general population: “*We did a number of pop-up clinics in the small town halls…and in some of our parks, as well as…the Giant Tiger parking lot…just to be able to grab people opportunistically…we did popups at the construction sites which had some good uptake there*” (FG8, P3, Physician).

Notably, some PHC teams undertook informal outreach activities to prevent having to discard unused vaccines. The QIDSS focus group explained, “*We’d have leftover vaccines, at one point…we were out on the sidewalk asking if people wanted vaccines, just so wasn’t getting wasted*” (FG2, P4, QIDSS). One of the IHP focus groups described how PHC conducted informal outreach activities to pedestrians on nearby streets: “*A few days we were in downtown [city name] and myself and some of the nurses were literally just walking the streets and asking people if they wanted vaccines*” (FG5, P4, Health Promoter). Similarly, informal outreach was utilized to engage hard-to-reach populations: “*There is this quite large population of…IV drug users…that maybe would be a higher risk of developing complications from COVID, homeless population…in the last two months I would always kind of walk the intersection, and just flag people down and ask them if they wanted to come in*” (FG5, P3, Pharmacist).

Some PHC teams did not participate in outreach activities because of administrative demands: “*The main reason for that is just because we had to register every patient manually in our system where we documented it there, and so it was pretty time-consuming to get those patients registered in, so not huge outreach beyond our patient population*” (FG5, P4, Health Promoter).

### PHC’s contributions being under-recognized

In all focus groups, some participants expressed concern and frustration that PHC’s contributions to the vaccination distribution was not widely recognized. Participants expressed concern about the perception that PHC was closed altogether: “*There was something in one news article…that primary care is closed! It stuck. People think that we were closed…that just stuck despite what was actually happening*” (FG5, P1, respiratory therapist). Despite being actively involved in vaccination distribution, some participants expressed frustration about the lack of recognition regarding PHC’s participation in intersectoral planning: “*They set up a vaccine rollout task force…Primary care was represented from…every [Family Health Team] in the region. So that’s why I kept getting very frustrated when I would hear people say that primary care was not, was being ignored – that was happening in some places, it was not happening in [Central Region Name]*” (FG8, P5, Physician). An IHP focus group also explained that there was a perception in the medical community that PHC was not actively participating in vaccination efforts: “*Within the medical community as well…I would hear comments of primary care being not as proactive, or not doing much, and physicians not seeing their patients, and it was so far from the truth that I think it was challenging…at the end of the day it does kind of weigh on you over time”* (FG5, P3, Pharmacist). Many focus groups expressed concerns that the extent of PHC’s involvement in the vaccination efforts went unrecognized: “*We did a couple clinic days at the arena…and we vaccinated the city staff, the firefighters [using] our own stuff, basically. And we ran through like double the amount of people that a Health Unit clinic would have ran through in the same amount of time…they don’t recognize that at all!*” (FG4, P3, Nurse).

## Discussion

This study provides unique insights demonstrating interprofessional PHC teams’ involvement with the COVID-19 vaccination distributions across various phases. The multiple roles that PHC providers hold in their community – such as family physicians in long-term care [32] - may have facilitated some PHC teams’ participation and leadership in the vaccine rollout. Consistent with recent research examining family physician leadership during the pandemic [33], participants in our study emphasized the leadership played by members of PHC teams during the pandemic that enabled community access to mass vaccination.

Similar to what was shared by participants in our study, some critics noted that PHC involvement was limited during the early phases of planning which may have led to the underutilization of PHC teams in the vaccine distribution [5, 10]. In Canada, early phases of the COVID-19 vaccination distribution were largely organized around public health, hospitals, and community pharmacies [5]. What is evident in our study were variations in how and when PHC providers and teams were involved in the vaccination rollouts, perhaps because of the emphasis on other healthcare sectors in early strategic planning. It has been suggested that other countries such as Israel and Singapore, employed a more cohesive national approach to strategizing and organizing the vaccine rollout, possibly because of their small geography and established communications systems [7].

PHC teams involvement in the distribution of the COVID-19 vaccination demonstrated a commitment to health equity [11, 20]. Early phases of vaccine rollout in Canada were accessible only through appointments, followed by vaccine efforts that extended to pop-up clinics and more specialized settings [11]. During the *priority groups* and *specialized settings* phases, PHC teams performed outreach activities to support the local community needs. Some focus group participants noted that their teams extended support to homebound, unhoused, and specialty cancer clinic populations. Our study demonstrates that the structure of care delivery in PHC teams enabled providers to identify patients who fit the criteria of priority populations and/or patients who were missed through mass vaccinations, such as those who were homebound or unhoused. It was during the government’s phase three call for PHC to start vaccinating that spurred teams to offer larger vaccination efforts. Although not a focus of provincial strategies for operationalizing vaccine efforts in early phases, activities pursued by PHC during those early phases helped facilitate vaccine reach in their communities.

An exceptional strength of our study was the range of interprofessional perspectives, which helped provide insights from across the multitude of providers and administrators who work within PHC teams and contributed to the vaccination rollouts. A limitation of our study is that it focused on one geographical setting in Ontario, Canada, so findings may not be applicable to all PHC settings. Findings represent one type of PHC model and may not represent the experiences of all PHC. It is also important to mention that while this study invited PHC teams to take part, individuals did have to reach out to express interest in participating which can result in a non-generalizable sample.

## Conclusion

Interprofessional PHC teams were involved in all phases of the COVID-19 vaccination efforts. PHC teams were visible in the community, provided leadership, and led outreach activities that enabled some of the most vulnerable populations in their communities to receive timely vaccinations.

### Electronic supplementary material

Below is the link to the electronic supplementary material.

Supplementary Material 1

Supplementary Material 2

## Data Availability

The datasets used and/or analyzed during the current study are available from the corresponding author on reasonable request.

## Abbreviations

AFHTO: Association of Family Health Teams of Ontario
IHPs: Interprofessional Healthcare Providers
PHC: Primary Health Care
