# Supplementary material for: A qualitative examination of primary care team’s participation in the distribution of the COVID-19 vaccination
Source: BMC Prim Care. 2024 Mar 14;25:85. doi: 10.1186/s12875-024-02327-2 (PMC10938813; doi:10.1186/s12875-024-02327-2)
Supplement: Supplementary file 1 — Supplementary Material 1 [file 12875_2024_2327_MOESM1_ESM.docx]

**Semi-Structured Interview Guide**

Executive Directors

1. **How and in what ways did you and your team contribute to the three different phases of Ontario’s vaccination strategy?**
   1. How were you engaged in regional and provincial planning and orchestration of vaccination strategies?
   2. In what settings did primary care provide and support vaccine distributions?
   3. What relationships did you engage in to support vaccine distributions?
      1. **Prompt:** Collaborations with community agencies, Ontario Health Teams, public health
   4. What do you think are the unique and ideal roles of primary care in relation to vaccination distribution?
   5. What are some of the challenges that you had to overcome?
   6. What challenges to do you anticipate as your team continues to follow Ontario’s vaccination strategy?
2. **How can primary care be best positioned to contribute to future vaccine distributions?**
   1. What would help primary care better prepare for future vaccine distributions?
   2. How can primary care collaborate with other sectors, like public health, to distribute vaccines?
   3. In what ways can primary care be utilized more effectively to respond to future pandemics?
   4. What are the most important opportunities for primary care to take advantage of in relation to vaccination distribution?

**Semi-Structured Interview Guide**

Healthcare Professionals (Nursing, Pharmacists, IHPs)

1. **How and in what ways did you and your team contribute to the three different phases of Ontario’s vaccination strategy? (i.e., long-term care, mass and pop-up clinics, opportunistic)**
   1. In what settings did primary care provide and support vaccine distributions?
   2. How did you engage patients and community partners to support vaccine distributions?
   3. What do you think are the unique and ideal roles of primary care in relation to vaccination distribution?
   4. What are some of the challenges that you had to overcome?
   5. What challenges to do you anticipate as your team continues to follow Ontario’s vaccination strategy?
2. **Please share your experiences with redeployment to support the vaccination strategy**
3. **How can primary care be best positioned to contribute to future vaccine distributions?**
   1. What would help primary care better prepare for future vaccine distributions?
   2. How can primary care collaborate with other sectors, like public health, to distribute vaccines?
   3. In what ways can primary care be utilized more effectively to respond to future pandemics?
   4. What are the most important opportunities for primary care to take advantage of in relation to vaccination distribution?
